# Defining the effects of prematurity on the lymphocyte transcriptome

**DOI:** 10.1186/1755-8166-7-S1-P87

**Published:** 2014-01-21

**Authors:** Soumyaroop Bhattacharya, Ravi Misra, Heidi Hyuck, Christopher Slaunwhite, Shannon Castiglione, Deanna Maffett, Anne Marie Reynolds, Gloria Pryhuber, Thomas Mariani

**Affiliations:** 1 Division of Neonatology, Department of Pediatrics, University of Rochester Medical Center, Rochester NY, USA; 2 Division of Neonatology, Department of Pediatrics, Women’s and Childrens Hospital, University at Buffalo, Buffalo NY, USA

## Background

We hypothesize that intrinsic and extrinsic factors associated with oxidative stress drive lymphocyte dysfunction contributing to lung disease in premature infants. Comprehensive transcriptomic assessment of CD8^+^ lymphocytes may identify biomarkers, and define disease-related mechanisms of chronic lung disease in premature infants.

## Methods

Peripheral blood samples were collected from premature infants at the time of hospital discharge at multiple sites and shipped to a central laboratory. Freshly purified PBMCs were isolated by Ficoll gradient centrifugation, sorted into individual lymphocyte cell types, and processed for total RNA. RNA isolated from CD8^+^ lymphocytes (n=79) was used for RNA-Seq analysis using the Illumina HiSeq2500. Sequences were aligned using the SHRiMP algorithm and expression values were summarized using HTSeq. Normalized gene expression data were analyzed for significant changes in expression using various statistical approaches. Ingenuity Pathway Analysis software was used for gene set interpretation.

## Results

Sufficient blood was collected to complete sorting from 137 of the 183 subjects recruited and discharged. The total lymphocyte frequency from the PBMC fraction was highly variable, with an average of 29.6±9.3%. Lymphocyte sub-type frequencies were also highly variable across subjects, with CD8^+^ cell averaging 5±2%. Total RNA yield from sorted cells varied according to cell type with CD8^+^ RNA averaging 177±140ng. RNA-Seq analysis using 1ng total RNA was completed for CD8^+^ cells from 79 subjects, generating an average of 11±5 million reads/sample with detection of 67±4% of the transcriptome. One-way ANOVA identified 147 genes that were differentially expressed among the 79 samples. A total of 47 genes were identified as differentially expressed between subjects born before 29-weeks and those born after 29-weeks of gestation. Pathways analysis of these genes identified mechanisms related to B and T cell signaling.

## Conclusion

These data demonstrate both the fidelity of our methodology and purity of the samples. Although we assessed a homogeneous cell type, our data includes substantial gene expression variability across subjects, and with respect to gestational age at birth. Our results support the feasibility of using these data and methods to identify biomarkers of, and mechanisms for, chronic respiratory morbidity following premature birth.

